# Supplementary material for: Individual Genomic Loci and mRNA Levels of Immune Biomarkers Associated with Pneumonia Susceptibility in Baladi Goats
Source: Vet Sci. 2023 Mar 1;10(3):185. doi: 10.3390/vetsci10030185 (PMC10051579; doi:10.3390/vetsci10030185)
Supplement: Supplementary file 1 [file vetsci-10-00185-s001.zip › vetsci-2215313-supplementary.pdf]

Supplementary:

Table S1. Immune gene oligonucleotide primer sequence used in PCR-DNA sequencing.

Gene	Forward	Reverse	Annealing temperature (°C)	Length of PCR product (bp)	Source
SLC11A1	5′-GTGCCCGAGTCTGCAGTCCTCA-3′	5′-AGAGTGGGATTCGTCCGGCTGAG-3′	62	523	Present research
CD-14	5′-GTGTGCTTGCCCTGCCTGCTGC-3′	5′-AGTGTGCTTGGGCAATGTTCAGC-3′	64	538	Present research
CCL2	5′-GCCAGTCCAGAGGCCAACAGCT-3′	5′-AGTCACGGAAGATAAACTGAA-3′	62	534	Present research
TLR1	5′-ATGCCTGACATCCTCTCACTATC-3′	5′-AGACAGTTCCAGACTCACTGTG-3′	60	471	Present research
TLR7	5′-ATCTTCCTCCCAGCTTACAGCTG-3′	5′- AGAATTATTTTCACAGGGTGTG-3′	60	398	Present research
TLR8	5′-GCTTCTGACCTCCCTTTTCCTGC-3′	5′- GTGTATCTGAATTGAAGCACCT-3′	62	799	Present research
TLR9	5′-ATGGGCCCCTACTGTGCCCCGC-3′	5′-TGCTGGTGCGGCTCAGCGACAG-3′	62	460	Present research
β defensin	5′-CAGCATGAGGCTCCATCACCTG-3′	5′-CGAAGGCGGCAGTTTCTGACT-3′	62	253	Present research
SP110	5′-TCCTGTTTTGCCCACTGGTATC-3′	5′-AGACACGGCAATAGCAGGGTC-3′	58	537	Present research
SPP1	5′-ATGAGAATTGCAGTGATTTGCT-3′	5′-CATTCACCAACTAAGCTGAGAA-3′	60	943	Present research
BP1	5′-ATGGCCAGCGGCCCCGGCAGT-3′	5′-AGGTTGCAGCTTGGAGGACACA -3′	62	645	Present research
A2M	5′-AACCGCAATACATGGTGCTGGT-3′	5′-GGTTTGTCTGTCTGGACAAAGAC -3′	58	325	Present research
ADORA3	5′-CTACTCACCTGGGAAGCTTCTC-3′	5′- CCTTGACCTTCTCGTCCATGAC-3′	64	521	Present research
CARD15	5′-ATGTGCGCACAAGATGCTTTTCA-3′	5′-GCTGCTGTGTCAGGTCCAGCAC -3′	62	394	Present research
IRF3	5′-ACTCCTGGGAAGGATAAGCCCG-3′	5′-AGGGCAGAAGACAGTCTGCTGG-3′	60	468	Present research

SCART1	5′-CTGAGATCAGGAATGTGAACGG-3′	5′-ACAGCAGGGACTCGTTGCCCGC-3′	62	475	Present research

SLC11A1 = Solute Carrier Family 11 Member 1; *CD14* = cluster of differentiation 14; *CCL2*= C-C motif ligand 2; *TLR1* = Toll-like receptor 1; *TLR7* = Toll-like receptor 7; *TLR8* = Toll-like receptor 8; *TLR 9*= Toll-like receptor 9; *β defensin* = beta defensin; *SP11 0*= SP110 Nuclear Body Protein; *SPP 1*= Secreted Phosphoprotein 1; *BP1* = bactericidal/permeability-increasing protein; *A2M* = Alpha-2-Macroglobulin; *ADORA3* = the adenosine A3 receptor; *CARD15* = caspase recruitment domain-containing protein 15; *IRF3* = interferon regulatory factor 3; *SCART1* = Scavenger Receptor Family Member Expressed On T Cells 1.

Table S2. Sequence of the oligonucleotide primers used in real-time PCR for immunological genes under investigation.

Gene	Primer	Product length (bp)	Annealing temperature (°C)	Accession number	Source
SLC11A1	F5′- GGACATCCGAGAAGCCAACA -3<br>R5′- TGGGAAAGATCGTCGCGTAG -3′	182	60	KX151137.1	Present research
CD-14	F5′- CAGCTGCAACAAGCTAAGCA -3<br>R5′- CGGTCTGGAATCATACGGGA -3′	144	59	DQ457090.1	Present research
CCL2	F5′- ACATTCTGCTTCGGCACTCA -3′<br>R5′- AGACCTTCATGTTGCAGCGA - 3′	200	60	XM_005693218.3	Present research
TLR1	F5′- ATGCCTGACATCCTCTCACT -3′<br>R5′- GAGGTTCAGAGTAGGGTGGC - 3′	162	58	NM_001285605.1	Present research
TLR7	F5′- CTTCTCTGTGTCTTCCAGGGTC-3′<br>R5′- CTACCGTTTCCTCACCTTGC - 3′	194	59	NM_001285543.2	Present research
TLR8	F5′- CCCGAAGCTATCCTTGCGAT -3′<br>R5′- CCACTCTGGGACTTGGCATT - 3′	217	60	GU936189.1	Present research
TLR9	F5′- TTCACCTTGGACCTGTCACG -3′<br>R5′- ATTAACCGCCTGGGAGATGC - 3′	117	60	EU747825.1	Present research
β defensin	F5′- CTTCCTGGTCCTGTCTGCTG -3′<br>R5′- GTTTCTAGGGCACCTGTCCG - 3′	106	62	DQ532360.1	Present research
SP110	F5′- TGCCCACGATTCTTTCACGA -3′<br>R5′- CGGGCATAAATGAGCGAGGA - 3′	108	62	XM_018058530.1	Present research
SPP1	F5′- CGCCCTTCCAGTTAAACCGA -3′<br>R5′- GCTTCTGAGATGGGTCAGGC - 3′	110	62	NM_001285667.1	Present research
BP1	F5′- TTCAGCATGGTTATTCGAGGA-3′<br>R5′- TAGAGATGCCCTCCACACTCA - 3′	178	58	NM_001314172.1	Present research
A2M	F5′- TGCAGTTTGCCCCTCACGAT-3′<br>R5′- CACCGTCTCATTCAGGTGGC- 3′	192	62	XM_018048527.1	Present research
ADORA3	F5′- TGAGGCTCTTCATTCTGCTCT-3′<br>R5′- CCAGTACTTGGGGTGGTCTT - 3′	143	58	XM_013962543.2	Present research
CARD15	F5′- TCCTGAGCACCTACGATGGA -3′<br>R5′- AAATGGTCACGGGTGCTGAA - 3′	157	60	NM_001314291.1	Present research
IRF3	F5′- ACATGACTCCCGAGCAACTG -3′<br>R5′- TGGCAGCCTTCCACAATGAT - 3′	103	60	JQ308793.1	Present research
SCART1	F5′- CCACTGGGACTTGGCAGAC -3′<br>R5′- TGAGATCAGGAATGTGAACGGG -3′	151	62	XR_001917340.1	Present research
ß. actin	F5′- GCCCCTACAGGTCATGTACTTC-3′<br>R5′- GCACAGCCTGGATGGCCACATAC -3′	113	60	AF481159.1	Present research

SLC11A1 = Solute Carrier Family 11 Member 1; *CD14* = cluster of differentiation 14; *CCL 2*= C-C motif ligand 2; *TLR1* = Toll-like receptor 1; *TLR7* = Toll-like receptor 7; *TLR8* = Toll-like receptor 8; *TLR9* = Toll-like receptor 9; *β defensin* = beta defensin; *SP110*= SP110 Nuclear Body Protein; *SPP1* = Secreted Phosphoprotein 1; *BP1* = bactericidal/permeability-increasing protein; *A2M* = Alpha-2-Macroglobulin; *ADORA3* = the adenosine A3 receptor; *CARD15* = caspase recruitment domain-containing protein 15; *IRF3* = interferon regulatory factor 3; *SCART1* = Scavenger Receptor Family Member Expressed On T Cells 1.

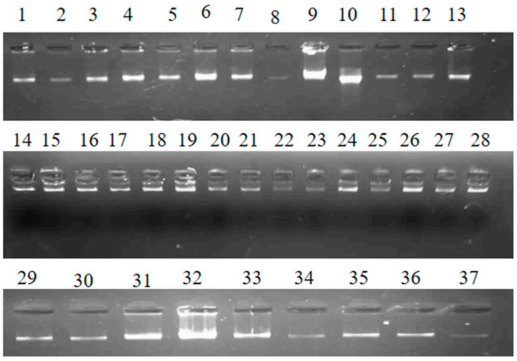

Figure S1: Representative gel electrophoresis for extracted DNA samples from the investigated does. Lanes from 1 to 37 are positive DNA samples.

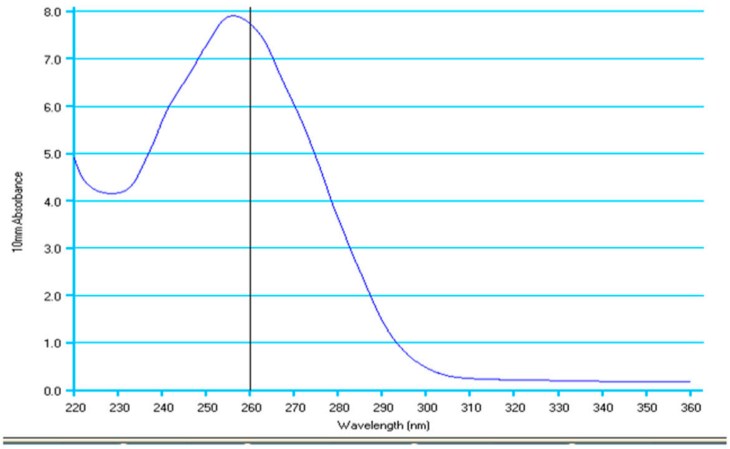

Figure S2: Nanodrop curve showing concentration of representative DNA extracted from the investigated does.

```
KX151137.1      GTGCCCGAGTCTGCAGTCCTCATGTCAGGTGACACGGGTACCCCAAACCAGGGAGGGACC 60
H               GTGCCCGAGTCTGCAGTCCTCATGTCAGGTGACACGGGTACCCCAAACCAGGGAGGGACC 60
P               GTGCCCGAGTCTGCAGTCCTCATGTCTGGTGACACGGGTACCCCAAACCAGGGAGGGACC 60
                ************************** *********************************
KX151137.1      AGATATGGCTCCATCTCCAGCCCACCCAGTCCAGGGCCACAGCAAGCACCTCCCGGAGGG 120
H               AGATATGGCTCCATCTCCAGCCCACCCAGTCCAGGGCCACAGCAAGCACCTCCCGGAGGG 120
P               AGATATGGCTCCATCTCCAGCCCACCCAGTCCAGGGCCACAGCAAGCACCTCCCGGAGGG 120
                ************************************************************
KX151137.1      ACCTACCTAAGTGAGAAGATCCCCATTCCGGATACAGAATCGGGTGCATTCAGCCTGCGG 180
H               ACCTACCTAAGTGAGAAGATCCCCATTCCGGATACAGAATCGGGTGCATTCAGCCTGCGG 180
P               ACCTACCTAAGTGAGAAGATCCCCATTCCGGATACAGAATCGGGTGCATTCAGCCTGCGG 180
                ************************************************************
KX151137.1      AAGCTGTGGGCCTTCACAGGGCCTGGATTCCTCATGAGCATAGCATTCCTGGACCCAGGA 240
H               AAGCTGTGGGCCTTCACAGGGCCTGGATTCCTCATGAGCATAGCATTCCTGGACCCAGGA 240
P               AAGCTGTGGGCCTTCACAGGGCCTGGATTCCTCATGAGCATAGCATTCCTGGACCCAGGA 240
                ************************************************************
KX151137.1      AACATCGAGTCGGATCTTCAGGCTGGGGCTGTGGCTGGATTCAAACTGCTCTGGGTGCTG 300
H               AACATCGAGTCGGATCTTCAGGCTGGGGCTGTGGCTGGATTCAAACTGCTCTGGGTGCTG 300
P               AACATCGAGTCGGATCTTCAGGCTGGGGCTGTGGCTGGATTCAAACTGCTCTGGGTGCTG 300
                ************************************************************
KX151137.1      CTGTGGGCCACAGTGTTGGGCTTGCTCTGCCAGCGACTGGCTGCCCGGCTGGGCGTGGTG 360
H               CTGTGGGCCACAGTGTTGGGCTTGCTCTGCCAGCGACTGGCTGCCCGGCTGGGCGTGGTG 360
P               CTGTGGGCCACAGTGTTGGGCTTGCTCTGCCAGCGACTGGCTGCCCGGCTGGGCGTGGTG 360
                ************************************************************
KX151137.1      ACAGGCAAGGACTTGGGAGAGGTCTGCCATCTCTACTACCCTAAGGTGCCCCGCACTCTC 420
H               ACAGGCAAGGACTTGGGAGAGGTCTGCCATCTCTACTACCCTAAGGTGCCCCGCATTCTC 420
P               ACAGGCAAGGACTTGGGAGAGGTCTGCCATCTCTACTACCCTAAGGTGCCCCGCACTCTC 420
                ******************************************************* ****
KX151137.1      CTCTGGCTGACCATCGAGCTAGCCATCGTGGGCTCGGACATGCAGGAAGTCATTGGCACA 480
H               CTCTGGCTGACCATCGAGCTAGCCATCGTGGGCTCGGACATGCAGGAAGTCATTGGCACA 480
P               CTCTGGCTGACCATCGAGCTAGCCATCGTGGGCTCGGACATGCAGGAAGTCATTGGCACA 480
                ************************************************************
KX151137.1      GCTATTGCATTCAGTCTGCTCTCAGCCGGACGAATCCCACTCT 523
H               GCTATTGCATTCAGTCTGCTCTCAGCCGGACGAATCCCACTCT 523
P               GCTATTGCATTCAGTCTGCTCTCAGCCGGACGAATCCCACTCT 523
                *******************************************
```

Figure S3: Comparison of healthy and pneumonia-affected Baladi goats using GenBank-sourced reference sequence gb|KX151137.1| and the *SLC11A1* gene (523-bp) demonstrating DNA sequence alignment.

```
NM_001361647.1      GTGTGCTTGCCCTGCCTGCTGCTGCTGCTGCTGCCGCCACTGCTGCGTGTGTCTGCGGAC      60
H                   GTGTGCTTGCCCTGCCTGCTGCTGCTGCTGCTGCCGCCACTGCTGCGTGTGTCTGCGGAC      60
P                   GTGTGCTTGCCCTGCCTGCTGCTGCTGCTGCTGCCGCCACTGCTGCGTGTGTCTGCGGAC      60
                    ************************************************************

NM_001361647.1      ACGACAGAGCCCTGCGAGCTGGACGACGACGATTTCCGCTGTGTCTGCAACTTCACGGAT      120
H                   ACGACAGAGCCCTGCGAGCTGGACGACGACGATTTCCGCTGTGTCTGCAACTTCACGGAT      120
P                   ACGACAGAGCCCTGCGAGCTGGACGACGACGATTTCCGCTGTGTCTGCAACTTCACGGAT      120
                    ************************************************************

NM_001361647.1      CCGAAGCCTGACTGGTCTAGCGCCGTTCAGTGTATGGTTGCCGTCGAGGTGGAGATCCGT      180
H                   CCGAAGCCTGACTGGTCTAGCGCCGTTCAGTGTATGGTTGCCGTCGAGGTGGAGATCCGT      180
P                   CCGAAGCCTGACTGGTCTAGCGCCGTTCAGTGTATGGTTGCCGTCGAGGTGGAGATCCGT      180
                    ************************************************************

NM_001361647.1      GGCGGCGGCCACAGCCTGGACCAGTTTCTCAAGGGAGCCAACACCGACCCGAAGCAGTAT      240
H                   GGCGGCGGCCACAGCCTGGACCAGTTTCTCAAGGGAGCCAACACCGACCCGAAGCAGTAT      240
P                   GGCGGCGGCCACAGCCTGGACCAGTTTCTCAAGGGAGCCAACACCGACCCGAAGCAGTAT      240
                    ************************************************************

NM_001361647.1      GCTGACACAATCAAGGCTCTGCGCGTTCGGCGACTCAAGCTGGGCGCTGCACAGGTTCCT      300
H                   GCTGACACAATCAAGGCTCTGCGCGTTCGGCGACTCAAGCTGGGCGCTGCACAGGTTCCT      300
P                   GCTGACACAATCAAGGCTCTGCGCGTTCGGCGACTCAAGCTGGGCGCTGCACAGGTTCCT      300
                    ************************************************************

NM_001361647.1      GCTCAGCTTCTGGTCGCCGTTCTGCGCGCGCTCGGGTACTCTCGTCTCAAGGAACTGACG      360
H                   GCTCAGCTTCTGGTCGCCGTTCTGCGCGCGCTCGGGTACTCTCGTCTCAAGGAACTGACG      360
P                   GCTCAGCTTCTGGTCGCCGTTCTGCGCGCGCTCGGGTACTCTCGTCTCAAGGAACTGACG      360
                    ************************************************************

NM_001361647.1      CTTGAGGACCTGGAGGTAACTGGCCCAACGCCCCCGGCGCCTCTGGAAGCCACTGGGCCT      420
H                   CTTGAGGACCTGGAGGTAACTGGCCCAACGCCCCCGGCGCCTCTGGAAGCCACTGGGCCT      420
P                   CTTGAGGACCTGGAGGTAACTGGCCCAACGCCCCCGGCGCCTCTGGAAGCCACTGGGCCT      420
                    ************************************************************

NM_001361647.1      GCGCTCACCACCCTCAGTCTCCGTAACGTGTCGTGGGCAACAGGAGGTGCCTGGCTCGGC      480
H                   GCGCTCACCACCCTCAGTCTCCGTAACGTGTCGTGGACAACAGGAGGTGCCTGGCTCGGC      480
P                   GCGCTCACCACCCTCAGTCTCCGTAACGTGTCGTGGGCAACAGGAGGTGCCTGGCTCGGC      480
                    ************************************ ***********************

NM_001361647.1      GAACTGCAGCAGTGGCTCAAGCCTGGGCTCAGGGCGCTGAACATTGCCCAAGCACACT        538
H                   GAACTGCAGCGGTGGCTCAAGCCTGGGCTCAGGGCGCTGAACATTGCCCAAGCACACT        538
P                   GAACTGCAGCAGTGGCTCAAGCCTGGGCTCAGGGCGCTGAACATTGCCCAAGCACACT        538
                    ********** ***********************************************
```

Figure S4: Comparison of healthy and pneumonia-affected Baladi goats using GenBank-sourced reference sequence gb|NM_001361647.1| and the *CD14* gene (538-bp) demonstrating DNA sequence alignment.

```
XM_004012471.4      GCCAGTCCAGAGGCCAACAGCTCCCACGCTGAAGCTTGAATCCTCTCGCTGCAACATGAA      60
H                   GCCAGTCCAGAGGCCAACAGCTCCCACGCTGAAGCTTGAATCCTCTCGCTGCAACATGAA      60
P                   GCCAGTCCAGAGGCCAACAGCTCCCACGCTGAAGCTTGAATCCTCTCGCTGCAACATGAA      60
                    ************************************************************

XM_004012471.4      GGTCTCCGCTGCTCTCCTCTGCCTGCTACTCACAGTAGCTGCCTTCAGCACCGAGGTGCT      120
H                   GGTCTCCGCTGCTCTCCTCTGCCTGCTACTCACAGTAGCTGCCTTCAGCACCGAGGTGCT      120
P                   GGTCTCCGCTGCTCTCCTCTGCCTGCTACTCACAGTAGCTGCCTTCAGCACCGAGGTGCT      120
                    ************************************************************

XM_004012471.4      CGCTCAGCCAGATGCAATTAACTCCCAAATCGCCTGCTGCTATAAATTCAATAAGAAGAT      180
H                   CGCTCAGCCAGATGCAATTAACTCCCAAATCGCCTGCTGCTATAAATTCAATAAGAAGAT      180
P                   CGCTCAGCCAGATGCAATTAACTCCCAAATCGCCTGCTGCTATAAATTCAATAAGAAGAT      180
                    ************************************************************

XM_004012471.4      CCCCATACAGAGGCTGACAAACTACAGAAGAGTCACCACCAGCAAGTGTCCCAAAGAAGC      240
H                   CCCCATACAGAGGCTGACAAACTACAGAAGAGTCACCACCAGCAAGTGTCCCAAAGAAGC      240
P                   CCCCATACAGAGGCTGACAAACTACAGAAGAGTCACCACCAGCAAGTGTCCCAAAGAAGC      240
                    ************************************************************

XM_004012471.4      TGTGATTTTCAAGACCATCCTAGGCAAAGAGTTTTGTGCAGACCCCAACCTGAAATGGGT      300
H                   TGTGATTTTCAAGACCATCCTGGGCAAAGAGTTTTGTGCAGACCCCAACCTGAAATGGGT      300
P                   TGTGATTTTCAAGACCATCCTAGGCAAAGAGTTTTGTGCAGACCCCAACCTGAAATGGGT      300
                    ********************* **************************************

XM_004012471.4      CCAGGACGCCATAAACCATCTCAACAAGAAAAACCAAACTCCGAAGCCTTGAGCACTCAC      360
H                   CCAGGACGCCATAAACCATCTCAACAAGAAAAACCAAACTCCGAAGCCTTGAGCACTCAC      360
P                   CCAGGACGCCATAAACCATCTCAACAAGAAAAACCAAACTCCGAAGCCTTGAGCACTCAC      360
                    ************************************************************

XM_004012471.4      TCCACAACCCAAGAATCTAAAGACAATCTGTGTTCTTCTGGCTTCCCCTAACTGCCCTCT      420
H                   TCCACAACCCAAGAATCTAAAGACAATCTGTGTTCTTCTGGCTTCCCCTAACTGCCCTCT      420
P                   TCCACAACCCAAGAATCTAAAGACAATCTGTGTTCTTCTGGCTTCCCCTAACTGCCCTCT      420
                    ************************************************************

XM_004012471.4      GATATTACCTTATTATAATTTTGAAGAGTATAACTTTATTGATGTGAACATGATGCCTTA      480
H                   GATATTACCTTATTATAATTTTGAAGAGTATAACTTTATTGATGTGAACATGATGCCTTA      480
P                   GATATTACCTTATTATAATTTTGAAGAGTATAACTTTATTGATGTGAACATGATGCCTCA      480
                    ********************************************************** *

XM_004012471.4      AGTAATGTTAATATTATTTAAGTTATTGATGCTTTCAGTTTATCTTCCGTGACT   534
H                   AGTAATGTTAATATTATTTAAGTTATTGATGCTTTCAGTTTATCTTCCGTGACT   534
P                   AGTAATGTTAATATTATTTAAGTTATTGATGCTTTCAGTTTATCTTCCGTGACT   534
                    ******************************************************
```

Figure S5: Comparison of healthy and pneumonia-affected Baladi goats using GenBank-sourced reference sequence gb|XM_004012471.4| and the *CCL2* gene (534-bp) demonstrating DNA sequence alignment.

```
KJ210567.1      ATGCCTGACATCCTCTCACTATCAAAGCTGAAAATTTTGATAATTTCTCATAATAGAATC 60
H               ATGCCTGACATCCTCTCACTATCAAAGCTGAAAATTTTGATAATTTCTCATAATAGAATC 60
P               ATGCCTGACATCCTCTCACTATCAAAGCTGAAAATTTTGATAATTTCTCATAATAGAATC 60
                ************************************************************

KJ210567.1      CAGTATCTTGACGGTGTTTTTAAATTCAACCACAGACTGGAATACTTGGATTTGTCCCAC 120
H               CAGTATCTTGACGGTGTTTTTAAATTCAACCACAGACTGGAATACTTGGATTTGTCCCAC 120
P               CAGTATCTTGACGGTGTTTTTAAATTCAACCACAGACTGGAATACTTGGATTTGTCCCAC 120
                ************************************************************

KJ210567.1      AACAATTTGGAGAAGATTTCTTGCCACCCTACTCTGAACCTCAAGCACTTAGACCTCTCA 180
H               AACAATTTGGAGAAGATTTCTTGCCACCCTACTCTGAACCTCAAGCACTTAGACCTCTCA 180
P               AACAATTTGGAGAAGATTTCTTGCCACCCTACTCTGAACCTCAAGCACTTAGACCTCTCA 180
                ************************************************************

KJ210567.1      TTTAATGCATTTGATGCCCTGCCCATATGCCAAGAGTTTGGCAACATGTCTCAACTAGAA 240
H               TTTAATGCATTTGATGCCCTGCCCATATGCCAAGAGTTTGGCAACATGTCTCAACTAGAA 240
P               TTTAATGCTTTTGATGCCCTGCCCATATGCCAAGAGTTTGGCAACATGTCTCAACTAGAA 240
                ******** ***************************************************

KJ210567.1      TTTCTGGGGTTGAGTGCCACACAGTTACAAAAATCCAGTGTGCAGTCAATCACTCGTTTG 300
H               TTTCTGGGGTTGAGTGCCACACAGTTACAAAAATCCAGTGTGCACTCAATCACTCGTTTG 300
P               TTTCTGGGGTTGAGTGCCACACAGTTACAAAAATCCAGTGTGCAGTCAATCACTCGTTTG 300
                ******************************************** ***************

KJ210567.1      CACATCAGCAAGGTTTTATTGGTCTTAGGAGATACTTATGGGGAAAGAGAAGATGCTGAG 360
H               CACATCAACAAGGTTTTATTGGTCTTAGGAGATACTTATGGGGAAAGAGAAGATGCTGAC 360
P               CACATCAGCAAGGTTTTATTGGTCTTAGGAGATACTTATGGGGAAAGAGAAGATGCTGAG 360
                ******* **************************************************** 

KJ210567.1      AGCCTTCAAGACCTTAAGACACAGAGCCTGCACGTTGTTTTCCCCACAGGAAAGGAATTC 420
H               AGCCTTCAAGACCTTAAGACACAGAGCCTGCACGTTGTTTTCCCCACAGGAAAGGAATTC 420
P               AGCCTTCAAGACCTTAAGACACAGAGCCTGCACTTTGTTTTCCCCACAGGAAAGGAATTC 420
                ********************************* **************************

KJ210567.1      CATTTTAATTTGGACGTGTCAGTCAGCACCACAGTGAGTCTGGAACTGTCT 471
H               CATTTTAATTTGGACGTGTCAGTCAGCACCACAGTGAGTCTGGAACTGTCT 471
P               CATTTTAATTTGGACGTGTCAGTCAGCACCACAGTGAGTCTGGAACTGTCT 471
                ***************************************************
```

Figure S6: Comparison of healthy and pneumonia-affected Baladi goats using GenBank-sourced reference sequence gb|KJ210567.1| and the *TLR1* gene (471-bp) demonstrating DNA sequence alignment.

```
GU289401.1      ATCTTCCTCCCAGCTTACAGCTGCTGAGCCTGGAGGCCAACAACATCTTCTTGATCATGA 60
H               ATCTTCCTCCCAGCTTACAGCTGCTGAGCCTGGAGGCCAACAACATCTTCTTGATCATGA 60
P               ATCTTCCTCCCAGCTTACAGCTGCTGAGCCTGGAGGCCAACAACATCTTCTTGATCATGA 60
                ************************************************************

GU289401.1      AGGAGAATCTAACAGAACTGGCCAACCTAGAAATACTCTACCTGGGCCAAAACTGTTACT 120
H               AGGAGAATCTAACAGAACTGGCCAACCTAGAAATACTCTACCTGGGCCAAAACTGTTACT 120
P               AGGAGAATCTAACAGAACTGGCCAACCTAGAAATACTCTACCTGGGCCAAAACTGTTACT 120
                ************************************************************

GU289401.1      ATCGTAACCCTTGTAATGTTTCATTTACTATCGAAAAAGATGCTTTCCTAAATATGAGAA 180
H               ATCGTAACCCTTGTAATGTTTCATTTACTATCGAAAAAGATGCTTTCCTAAATATGAGAA 180
P               ATCGTAACCCTTGTAATGTTTCATTTACTATCGAAAAAGATGCTTTCCTAAATATGAGAA 180
                ************************************************************

GU289401.1      ATTTAAAATTGCTCTCCCTAAAAGATAACAATATCTCAGCTGTCCCCACTGTTTTGCCAT 240
H               ATTTAAAATTGCTCTCCCTAAAAGATAACAATATCTCAGCTGTCCCCACTGTTTTGCCAT 240
P               ATTTAAAATTGCTCTCCCTAAAAGATAACAATATCTCAGCTGTCCCCACTGTTTTGCCAT 240
                ************************************************************

GU289401.1      CTAGTTTGACAGAACTCTATCTTTACAATAACATCATTACAAAAATCCAAGAAGATGATT 300
H               CTAGTTTGACAGAACTCTATCTTTACAATAACATCATTACAAAAATCCAAGAAGATGATT 300
P               CTAGTTTGACAGAACTCTATCTTTACAATAACATCATTACAAAAATCCAAGAAGATGATT 300
                ************************************************************

GU289401.1      TTAATAACCTCAGTCAACTACAAGTTCTTGATCTGAGTGGAAATTGCCCTCGTTGTTATA 360
H               TTAATAACCTCAGTCAACTACAAGTTCTTGATCTGAGTGGAAATTGTCCTCGTTGTTATA 360
P               TTAATAACCTCAGTCAACTACAAGTTCTTGATCTGAGTGGAAATTGCCCTCGTTGTTATA 360
                ********************************************** *************

GU289401.1      ATGTTCCATTTCCTTGCACACCCTGTGAAAATAATTCT    398
H               ATGTTCCATTTCCTTGCACACCCTGTGAAAATAATTCT    398
P               ATGTTCCATTTCCTTGCACACCCTGTGAAAATAATTCT    398
                **************************************
```

Figure S7: Comparison of healthy and pneumonia-affected Baladi goats using GenBank-sourced reference sequence gb|GU289401.1| and the *TLR7* gene (398-bp) demonstrating DNA sequence alignment.

```
GU936189.1      GCTTCTGACCTCCCTTTTCCTGCTCATCTCCGATTCCTGTGAGTTCTTCACTGAAGCCAG 60
H               GCTTCTGACCTCCCTTTTCCTGCTCATCTCCGATTCCTGTGAGTTCTTCACTGAAGCCAG 60
P               GCTTCTGACCTCCCTTTTCCTGCTCATCTCCGATTCCTGTGAGTTCTTCACTGAAGCCAG 60
                ************************************************************

GU936189.1      TTATCCCCGAAGCTATCCTTGCGATGTGAAAAACAATAATGGCTCTTTTATTGCAGAATG 120
H               TTATCCCCGAAGCTATCCTTGCGATGTGAAAAACAATAATGGCTCTTTTATTGCAGAATG 120
P               TTATCCCCGAAGCTATCCTTGCGATGTGAAAAACAATAATGGCTCTTTTATTGCAGAATG 120
                ************************************************************

GU936189.1      TAACAGTCGTCGATTACAGGAAGTACCCCAAACAGTGGACAAAGCTGTGACAGAAGTAGA 180
H               TAACAGTCGTCGATTACAGGAAGTACCCCAAACAGTGGACAAAGCTGTGACAGAAGTAGA 180
P               TAACAGTCGTCGATTACAGGAAGTACCCCAAACAGTGGACAAAGCTGTGACAGAAGTAGA 180
                ************************************************************

GU936189.1      CCTGTCTGATAATTTCATCACACGTATAACGAATGAATCCTTTCAAGGGCTGCAAAATCT 240
H               CCTGTCTGATAATTTCATCACACGTATAACGAATGAATCCTTTCAAGGGCTGCAAAATCT 240
P               CCTGTCTGATAATTTCATCACACGTATAACGAATGAATCCTTTCAAGGGCTGCAAAATCT 240
                ************************************************************

GU936189.1      GACTAAAATCAACCTGAACCATAATGCCAAGTCCCAGAGTGGAAATCCTGCTGTAAAGAA 300
H               GACTAAAATCAACCTGAACCATAATGCCAAGTCCCAGAGTGGAAATCCTGCTGTAAAGAA 300
P               GACTAAAATCAACCTGAACCATAATGCCAAGTCCCAGAGTGGAAATCCTGCTGTAAAGAA 300
                ************************************************************

GU936189.1      AGCTATGACTATTACAGACGGGGCATTTCTCAACCTCAAACACCTAAGGGAGTTGCTGCT 360
H               AGCTATGACTATTACAGACGGGGCATTTCTCAACCTCAAACACCTAAGGGAGTTGCTGCT 360
P               AGCTATGACTATTACAGACGGGGCATTTCTCAACCTCAAACACCTAAGGGAGTTGCTGCT 360
                ************************************************************

GU936189.1      GGAAGACAACCAGTTACAAGAAATACCGGCTGGTTTGCCAGAATCTTTGAAAGAACTTAG 420
H               GGAAGACAACCAGTTACAAGAAATACCGGCTGGTTTGCCAGAATCTTTGAAAGAACTTAG 420
P               GGAAGACAACCAGTTACAAGAAATACCGGCTGGTTTGCCAGAATCTTTGAAAGAACTTAG 420
                ************************************************************

GU936189.1      TCTAATTCAAAACAATATAATTACGTTAACGAAAAAGAACACTTCTGGACTTGGGAACCT 480
H               TCTAATTCAAAACAATATAATTACGTTAACGAAAAAGAACACTTCTGGACTTGGGAACCT 480
P               TCTAATTCAAAACAATATAATTACGTTAACGAAAAAGAACACTTCTGGACTTGGGAACCT 480
                ************************************************************

GU936189.1      GGAACGTCTCTATTTGGGCTGGAACTGTTATTTTGCTTGTAGTAAAAAATTTAGCATAGA 540
H               GGAACGTCTCTATTTGGGCTGGAACTGTTATTTTGCTTGTAGTAAAAAATTTAGCATAGA 540
P               GGAACGTCTCTATTTGGGCTGGAACTGTTATTTTGCTTGTAGTAAAAAATTTAGCATAGA 540
                ************************************************************

GU936189.1      AAATGGAGCATTCCAAAACCTTACCAAGTTGAAGGTGCTGTCATTATCTTTTAATCCCCT 600
H               AAATGGAGCATTCCAAAACCTTACCAAGTTGAAGGTGCTGTCATTATCTTTTAATCCCCT 600
P               AAATGGAGCATTCCAAAACCTTACCAAGTTGAAGGTGCTGTCATTATCTTTTAATCCCCT 600
                ************************************************************

GU936189.1      TCACAGCGTGCCACCGAGTCTGCCAAGCTCGCTAACAGAACTCTACCTTAGCAATACCCA 660
H               TCACAGCGTGCCACCGAGTCTGCCAAGCTCGCTAACAGAACTCTACCTTAGCAATACCCA 660
P               TCACAGCGTGCCACCGAGTCTGCCAAGCTCGCTAACAGAACTCTACCTTAGCAATACCCA 660
                ************************************************************
GU936189.1      TATTGGAAACGTCAGTGAAGAAGACTTCAAGGAACTGAGCAATTTAAGGGTACTAGATTT 720
H               TATTGGAAACGTCAGTGAAGAAGACTTCAAGGAACTGAGCAATTTAAGAGTACTAGATTT 720
P               TATTGGAAACGTCAGTGAAGAAGACTTCAAGGAACTGAGCAATTTAAGGGTACTAGATTT 720
                ************************************************ ***********

GU936189.1      AAGTGGAAACTGCCCGAGATGTTTTAACGCTCCGTTTCCCTGTGTACCTTGCCAAGGAGG 780
H               AAGTGGAAACTGCCCGAGATGTTTTAACGCTCCGTTTCCCTGTGTACCTTGCCAAGGAGG 780
P               AAGTGGAAACTGCCCGAGATGTTTTAACGCTCCGTTTCCCTGTGTACCTTGCCAAGGAGG 780
                ************************************************************

GU936189.1      TGCTTCAATTCAGATACAC   799
H               TGCTTCAATTCAGATACAC   799
P               TGCTTCAATTCAGATACAC   799
                *******************
```

Figure S8: Comparison of healthy and pneumonia-affected Baladi goats using GenBank-sourced reference sequence gb|GU936189.1| and the *TLR8* gene (799-bp) demonstrating DNA sequence alignment.

```
EU747825.1      ATGGGCCCCTACTGTGCCCCGCACCCCCTTTCTCTTCTGGTGCAGGCAGCGGCGCTGGCA 60
H               ATGGGCCCCTACTGTGCCCCGCACCCCCTTTCTCTTCTGGTGCAGGCAGCGGCGCTGGCA 60
P               ATGGGCCCCTACTGTGCCCCGCACCCCCTTTCTCTTCTGGTGCAGGCAGCGGCGCTGGCA 60
                ************************************************************
EU747825.1      GCAGCCCTGGCCCAGGGCACCCTGCCTGCCTTCCTGCCCTGTGAGCTCCAGCCCCGGGGT 120
H               GCAGCCCTGGCCCAGGGCACCCTGCCTGCCTTCCTGCCCTGTGAGCTCCAGCCCCGGGGT 120
P               GCAGCCCTGGCCCAGGGCACCCTGCCTGCCTTCCTGCCCTGTGAGCTCCAGCCCCGGGGT 120
                ************************************************************
EU747825.1      CAGGTGAACTGCAACTGGCTGTTCCTGAAGTCTGTGCCGCGCTTTTCGGCCGGAGCCCCC 180
H               CAGGTGAACTGCAACTGGCTGTTCCTGAAGTCTGTGCCGCGCTTTTCGGCCGGAGCCCCC 180
P               CAGGTGAACTGCAACTGGCTGTTCCTGAAGTCTGTGCCGCGCTTTTCGGCCGGGGCCCCC 180
                ***************************************************** ******
EU747825.1      CGGGCCAATGTCACCAGCCTCTCCTTAATCTCCAACCGCATCCACCACTTGCACGACTCT 240
H               CGGGCCAATGTCACCAGCCTCTCCTTAATCTCCAACCGCATCCACCACTTGCACGACTCT 240
P               CGGGCCAATGTCACCAGCCTCTCCTTAATCTCCAACCGCATCCACCACTTGCACGACTCT 240
                ************************************************************
EU747825.1      GACTTCGTCCACCTGTCCAACCTGCGGGTCCTCAACCTCAAGTGGAACTGCCCGCCGGCC 300
H               GACTTCGTCCACCTGTCCAACCTGCGGGTCCTCAACCTCAAGTGGAACTGCCCGCCGGCC 300
P               GACTTCGTCCACCTGTCCAACCTGCGGGTCCTCAACCTCAAGTGGAACTGCCCGCCGGCC 300
                ************************************************************
EU747825.1      GGCCTCAGCCCCATGCACTTCCCCTGCCGCATGACCATTGAGCCCAACACCTTCCTGGCC 360
H               GGCCTCAGCCCCATGCACTTCCCCTGCCGCATGACCATTGAGCCCAACACCTTCCTGGCC 360
P               GGCCTCAGCCCCATGCACTTCCCCTGCCGCATGACCATTGAGCCCAACACCTTCCTGGCT 360
                *********************************************************** 
EU747825.1      GTGCCCACCCTGGAGGAGCTGAACCTGAGCTACAATGGCATCACGACCGTGCCTGCCCTG 420
H               GTGCCCACCCTGGAGGAGCTGAACCTGAGCTACAATGGCATCACGACCGTGCCTGCCCTG 420
P               GTGCCCACCCTGGAGGAGCTGAACCTGAGCTACAATGGCATCACGACCGTGCCTGCCCTG 420
                ************************************************************
EU747825.1      CCCAGTTCTCTCGTATCCCTGTCGCTGAGCCGCACCAGCA  460
H               CCCAGTTCTCTCGTATCCCTGTCGCTGAGCCGCACCAGCA  460
P               CCCAGTTCTCTCGTATCCCTGTCGCTGAGCCGCACCAGCA  460
                ****************************************
```

Figure S9: Comparison of healthy and pneumonia-affected Baladi goats using GenBank-sourced reference sequence gb|EU747825.1| and the *TLR9* gene (460-bp) demonstrating DNA sequence alignment.

```
DQ532360.1      CAGCATGAGGCTCCATCACCTGCTCCTCGCGCTCTTCTTCCTGGTCCTGTCTGCTGGGTC 60
H               CAGCATGAGGCTCCATCACCTGCTCCTCGCGCTCTTCTTCCTGGTCCTGTCTGCTGGGTC 60
P               CAGCATGAGGCTCCATCACCTGCTCCTCGTGCTCTTCTTCCTGGTCCTGTCTGCTGGGTC 60
                ***************************** ******************************

DQ532360.1      AGGATTTACTCAAGGAATAATAAATCATCGAAGCTGCCATAGGATTAAAGGCGTCTGTGC 120
H               AGGATTTACTCAAGGAATAATAAATCATCGAAGCTGCCATAGGATTAAAGGCGTCTGTGC 120
P               AGGATTTACTCAAGGAATAATAAATCATCGAAGCTGCCATAGGAATAAAGGCGTCTGTGC 120
                ******************************************** ***************

DQ532360.1      GCCGGACAGGTGCCCTAGAAACATGAGACAGATTGGCACCTGTTTCGGGCCCCCAGTAAA 180
H               GCCGGACAGGTGCCCTAGAAACATGAGACAGATTGGCACCTGTTTCGGGCCCCCAGTAAA 180
P               GCCGGACAGGTGCCCTAGAAACATGAGACAGATTGGCACCTGTTTCGGGCCCCCAGTAAA 180
                ************************************************************

DQ532360.1      ATGCTGCAGAAAGAAGTAACAGAAGGCGAAGACGCGGCCGGGACCGATGCGGAGTCAGAA 240
H               ATGCTGCAGAAAGAAGCAACAGAAGGCGAAGACGCGGCCGGGACCGATGCGGAGTCAGAA 240
P               ATGCTGCAGAAAGAAGTAACAGAAGGCGAAGACGCGGCCGGGACTGATGCGGAGTCAGAA 240
                **************** *************************** ***************

DQ532360.1      ACTGCCGCCTTCG       253
H               ACTGCCGCCTTCG       253
P               ACTGCCGCCTTCG       253
                *************
```

Figure S10: Comparison of healthy and pneumonia-affected Baladi goats using GenBank-sourced reference sequence gb|DQ532360.1| and the β defensin gene (253-bp) demonstrating DNA sequence alignment.

```
XM_013973239.2      TCCTGTTTTGCCCACTGGTATCTCTCTGCCCTTTTCTCAGTCTCCCAGGAGCCACCCCAG      60
H                   TCCTGTTTTGCCCACTGGTATCTCTCTGCCCTTTTCTCAGTCTCCCAGGAGCCACCCCAG      60
P                   TCCTGTTTTGCCCACTGGTATCTCTCTGCCCTTTTCTCAGTCTCCCAGGAGCCACCCCAG      60
                    ************************************************************

XM_013973239.2      AACAGCCCCTGGATCTGGAAGGGGACACGAACACCTAGTCATCTCAGCATCCAGGAAAGC      120
H                   ACCAGCCCCTGGATCTGGAAGGGGACACGAACACCTAGTCATCTCAGCATCCAGGAAAGC      120
P                   AACAGCCCCTGGATCTGGAAGGGGACACGAACACCTAGTCATCTCAGCATCCAGGAAAGC      120
                    * **********************************************************

XM_013973239.2      CACCATGACCAGGGCCTTGGAAGAGGCTCTTCTTCAGCACTTCATACATCAGAAGCTGGA      180
H                   CACCATGACCAGGGCCTTGGAAGAGGCTCTTCTTCAGCACTTCATACATCAGAAGCTGGA      180
P                   CACCATGACCAGGGCCTTGGAAGAGGCTCTTCTTCAGCACTTCATACATCAGAAGCTGGA      180
                    ************************************************************

XM_013973239.2      GATCGCTTATGCCATCAACAAGCCATTTCCCTTCTTCGAAGGCCTCCGAGACAACTCCTT      240
H                   GATTGCTTATGCCATCAACAAGCCATTTCCCTTCTTCGAAGGCCTCCGAGACAACTCCTT      240
P                   GATCGCTTATGCCATCAACAAGCCATTTCCCTTCTTCGAAGGCCTCCGAGACAACTCCTT      240
                    *** ********************************************************

XM_013973239.2      CATCACGGAGAGACTGTACAGGGAATCTATGGAAGCCTGGGAAAATCTGGTCCCTTTATG      300
H                   CATCACGGAGAGACTGTACAGGGAATCTATGGAAGCCTGGGAAAATCTGGTCCCTTTATG      300
P                   CATCACGGAGAGACTGTACAGGGAATCTATGGAAGCCTGGGAAAATCTGGTCCCTTTATG      300
                    ************************************************************

XM_013973239.2      CAGAGTTGTGTACAACATTCTTACCCAACTGGAGAAGACATTTAGTCTATCATTTCTGAA      360
H                   CAGAGTTGTGTACAACATTCTTACCCAACTGGAGAAGACATTTAGTCTATCATTTCTGAA      360
P                   CAGAGTTGTGTACAACATTCTTACCCAACTGGAGAAGACATTTAGTCTATCATTTCTGAA      360
                    ************************************************************

XM_013973239.2      GATATTGTTCAGCCGAATTAACCTGAAAGCATACCCCAACCTGATAACGGCTCTCAGTAG      420
H                   GATATTGTTCAGCCGAATTAACCTGAAAGCATACCCCAACCTGATAACGGCTCTCAGTAG      420
P                   GATATTGTTCAGCCGAATTAACCTGAAAGCATACCCCAACCTGATAACGGCTCTCAGTAG      420
                    ************************************************************

XM_013973239.2      CTTCACAAGAGTCGTCACTTCCCATGGAGGCTGGAGCAGAAACACAACCATCCCTCTTGA      480
H                   CTTCACAAGAGTCGTCACTTCCCATGGAGGCTGGAGCAGAAACACAACCATCCCTCTTGA      480
P                   CTTCACAAGAGTCGTCACTTCCCATGGAGGCTGGAGCAGAAACACAACCATCCCTCTTGA      480
                    ************************************************************

XM_013973239.2      AGCCCCAGCCAACCCAGCAGGAAAGAGCTCCTCGAGGACCCTGCTATTGCCGTGTCT         537
H                   AGCCCCAGCCAACCCAGCAGGAAAGAGCTCCTCGAGGACCCTGCTATTGCCGTGTCT         537
P                   AGCCCCAGCCAACCCAGCAGGAAAGAGCTCCTCGAGGACCCTGCTATTGCCGTGTCT         537
                    *********************************************************
```

Figure S11: Comparison of healthy and pneumonia-affected Baladi goats using GenBank-sourced reference sequence gb|XM_013973239.2|and the *SP110* gene (537-bp) demonstrating DNA sequence alignment.

```
NM_001285667.1      ATGAGAATTGCAGTGATTTGCTTCTGCCTCTTGGGCATTGCCTCCGCCCTTCCAGTTAAA      60
H                   ATGAGAATTGCAGTGATTTGCTTCTGCCTCTTGGGCATTGCCTCCGCCCTTCCAGTTAAA      60
P                   ATGAGAATTGCAGTGATTTGCTTCTGCCTCTTGGGCATTGCCTCCGCCCTTCCAGTTAAA      60
                    ************************************************************

NM_001285667.1      CCGACCAGTTCTGGCAGCTCTGAGGAAAAGCTGCTTAACAACAAATACCCAGATGCTGTA      120
H                   CCGACCAGTTCTGGCAGCTCTGAGGAAAAGCTGCTTAACAACAAATACCCAGATGCTGTA      120
P                   CCGACCAGTTCTGGCAGCTCTGAGGAAAAGCTGCTTAACAACAAATACCCAGATGCTGTA      120
                    ************************************************************

NM_001285667.1      GCCACATGGCTAAAGCCTGACCCATCTCAGAAGCAGACTTTCCTAGAACCCCAGAATTCT      180
H                   GCCACATGGCTAAAGCCTGACCCATCTCAGAAGCAGACTTTCCTAGAACCCCAGAATTCT      180
P                   GCCACATGGCTAAAGCCTGACCCATCTCAGAAGCAGACTTTCCTAGAACCCCAGAATTCT      180
                    ************************************************************

NM_001285667.1      GTGTCCTCTGAGGAAACTGATGACAACAAACAAAATACCCTCCCGAGTAAGTCCAATGAA      240
H                   GTGTCCTCTGAGGAAACTGATGACAACAAACAAAATACCCTCCCGAGTAAGTCCAATGAA      240
P                   GTGTCCTCTGAGGAAACTGATGACAACAAACAAAATACCCTCCCGAGTAAGTCCAATGAA      240
                    ************************************************************

NM_001285667.1      AGCCCTGAGCAAACAGACGATCTAGATGATGATGATGAAAACAGCCAGGAAGTCAACTCT      300
H                   AGCCCTGAGCAAACAGACGATCTAGATGATGATGATGAAAACAGCCAGGAAGTCAACTCT      300
P                   AGCCCTGAGCAAACAGACGATCTAGATGATGATGATGAAAACAGCCAGGAAGTCAACTCT      300
                    ************************************************************

NM_001285667.1      GATGACTCCGACGATGCTAACCCTGATGACTCTGACCATTCCAACGAGTCTCACCATTCT      360
H                   GATGACTCCGACGATGCTAACCCTGATGACTCTGACCATTCCAACGAGTCTCACCATTCT      360
P                   GATGACTCCGACGATGCTAACCCTGATGACTCTGACCATTCCAACGAGTCTCACCATTCT      360
                    ************************************************************

NM_001285667.1      GATGAATCTGATGAAGCTGATTTTCCCACTGACATTCCAACAATCGCAGTTTTCACTCCA      420
H                   GATGAATCTGATGAAGCTGATTTTCCCACTGACATTCCAACAATCGCAGTTTTCACTCCA      420
P                   GATGAATCTGATGAAGCTGATTTTCCCACTGACATTCCAACAATCGCAGTTTTCACTCCA      420
                    ************************************************************

NM_001285667.1      CCTTTCCCTACGGAAAGCACAAATGATGGCCGAGGTGATAGTGTGGCTTATGGACTGAAG      480
H                   CCTTTCCCTACGGAAAGCACAAATGATGGCCGAGGTGATAGTGTGGCTTATGGACTGAAG      480
P                   CCTTTCCCTACGGAAAGCACAAATGATGGCCGAGGTGATAGTGTGGCTTATGGACTGAAG      480
                    ************************************************************

NM_001285667.1      TCAAAATCGAAGAAGTTCCGCCGATCTAACGTTGAGAGTCCAGATGCCACAGAGGAGGAC      540
H                   TCAAAATCGAAGAAGTTCCGCCGATCTAACGTTGAGAGTCCAGATGCCACAGAGGAGGAC      540
P                   TCAAAATCGAAGAAGTTCCGCCGATCTAACGTTGAGAGTCCAGATGCCACAGAGGAGGAC      540
                    ************************************************************

NM_001285667.1      TTCACATCACACATAGAGAGTGAGGAGATGCATGATGCACCTAAGAAGACGAGTCAGCTG      600
H                   TTCACATCACACATAGAGAGTGAGGAGATGCATGATGCACCTAAGAAGACGAGTCAGCTG      600
P                   TTCACATCACACATAGAGAGTGAGGAGATGCATGATGCACCTAAGAAGACGAGTCAGCTG      600
                    ************************************************************

NM_001285667.1      ACTGACCACAGCGAAGAAACCAACAGTGACGAGCTTTCCAAAGAACTCACACCAAAGGCC      660
H                   ACTGACCACAGCGAAGAAACCAACAGTGACGAGCTTTCCAAAGAACTCACACCAAAGGCC      660
P                   ACTGACCACAGCGAAGAAACCAACAGTGACGAGCTTTCCAAAGAACTCACACCAAAGGCC      660
                    ************************************************************

NM_001285667.1      CAGGAGGAGAGCAAGCATTCCAATCGGATCGAGAGTCAGGAAAATTCCAAACTCAGCCAA      720
H                   CAGGAGGAGAGCAAGCATTCCAATCGGATCGAGAGTCAGGAAAATTCCAAACTCAGCCAA      720
P                   CAGGAGGAGAGCAAGCATTCCAATCGGATCGAGAGTCAGGAAAATTCCAAACTCAGCCAA      720
                    ************************************************************

NM_001285667.1      GAATTCCATAGCCTTGAAGACAAGCTAGACCTAGATCATAAGAGTGAAGAAGACAAGCGC      780
H                   GAATTCCATAGCCTTGAAGACAAGCTAGACCTAGATCATAAGAGTGAAGAAGACAAGCGC      780
P                   GAATTCCATAGCCTTGAAGACAAGCTAGACCTAGATCATAAGAGTGAAGAAGACAAGCGC      780
                    ************************************************************

NM_001285667.1      CTGAAAATCCGCATTTCTCATGAATTAGATAGTGCCTCTTCTGAGGTCAACTGAAAGGAG      840
H                   CTGAAAATCCGCATTTCTCATGAATTAGATAGTGCCTCTTCTGAGGTCAACTGAAAGGAG      840
P                   CTGAAAATCCGCATTTCTCATGAATTAGATAGTGCCTCTTCTGAGGTCAACTGAAAGGAG      840
                    ************************************************************

NM_001285667.1      AAAATACAAGTTCTTACTTTGCTTTTAGTAAAAAGAAAAGGATATGTTAAAGCAGGGTGG      900
H                   AAAATACAAGTTCTTACTTTGCTTTTAGTAAAAAGAAAAGGATATGTTAAAGCAGGGTGG      900
P                   AAAATACAAGTTCTTATTTTGCTTTTAGTAAAAAGAAAAGGATATGTTAAAGCAGGGTGG      900
                    **************** *******************************************

NM_001285667.1      GAGACAATATGAAATGCGTATTTCTCAGCTTAGTTGGTGAATG      943
H                   GAGACAATATGAAATGCGTATTTCTCAGCTTAGTTGGTGAATG      943
P                   GAGACAATATGAAATGCGTATTTCTCAGCTTAGTTGGTGAATG      943
                    *******************************************
```

Figure S12: Comparison of healthy and pneumonia-affected Baladi goats using GenBank-sourced reference sequence gb|NM_001285667.1| and the *SPP1* gene (943-bp) demonstrating DNA sequence alignment.

```
KC894598.1      ATGGCCAGCGGCCCCGGCAGTGTGCGGAGGTGGGCAACCCTGGTGGTGCTGGTCGCCCTG 60
H               ATGGCCAGCGGCCCCGGCAGTGTGCGGAGGTGGGCAACCCTGGTGGTGCTGGTCGCCCTG 60
P               ATGGCCAGCGGCCCCGGCAGTGTGCGGAGGTGGGCAACCCTGGTGGTGCTGGTCGCCCTG 60
                ************************************************************
KC894598.1      GGCACGGCTGTGACAACGACGACCAACCCCGGCATCGTGGCCAGGGTCACCCAGAAAGGC 120
H               GGCACGGCTGTGACGACGACGACCAACCCCGGCATCGTGGCCAGGGTCACCCAGAAAGGC 120
P               GGCACGGCTGTGACAACGACGACCAACCCCGGCATCGTGGCCAGGGTCACCCAGAAAGGC 120
                ************** *********************************************
KC894598.1      CTGGACTACGCCTGCCAGCAGGGAGTGCTTACTCTGCAGAAAGAGTTGGAGAAGATAACA 180
H               CTGGACTACGCCTGCCAGCAGGGAGTGCTTACTCTGCAGAAAGAGTTGGAGAAGATAACA 180
P               CTGGACTACGCCTGCCAGCAGGGAGTGCTTACTCTGCAGAAAGAGTTGGAGAAGATAACA 180
                ************************************************************
KC894598.1      ATTCCCACTTTCTCAGGAAACTTTAAGATAAAATACCTCGGGAAAGGGCAATACAGCTTC 240
H               ATTCCCACTTTCTCAGGAAACTTTAAGATAAAATACCTCGGGAAAGGGCAATACAGCTTC 240
P               ATTCCCACTTTCTCAGGAAACTTTAAGATAAAATACCTCGGGAAAGGGCAATACAGCTTC 240
                ************************************************************
KC894598.1      TTCAGCATGGTTATTCGAGGATTCAATCTTCCCAATTCCCAGATCAGACCGTTGCCGGAT 300
H               TTCAGCATGGTTATTCAAGGATTCAATCTTCCCAATTCCCAGATCAGACCGTTGCCGGAT 300
P               TTCAGCATGGTTATTCGAGGATTCAATCTTCCCAATTCCCAGATCAGACCGTTGCCGGAT 300
                **************** *******************************************
KC894598.1      GAGGGCCTTGATCTCTCTATCAGAGATGCCAGTATCAAGATCAGAGGAAAATGGAAGGCA 360
H               GAGGGCCTTGATCTCTCTATCAGAGATGCCAGTATCAAGATCAGAGGAAAATGGAAGGCA 360
P               GAGGGCCTTGATCTCTCTATCAGAGATGCCAGTATCAAGATCAGAGGAAAATGGAAGGCA 360
                ************************************************************
KC894598.1      CGAAAGAACTTCATCAAACTCGGCGGCAACTTTGACCTGAGTGTGGAGGGCATCTCTATT 420
H               CGAAAGAACTTCATCAAACTCGGCGGCAACTTTGACCTGAGTGTGGAGGGCATCTCTATT 420
P               CGAAAGAACTTCATCAAACTCGGCGGCAACTTTGACCTGAGTGTGGAGGGCATCTCTATT 420
                ************************************************************
KC894598.1      TCGGCGGGTCTGAGTCTGGGCTATGACCCTGACTCAGGCCACTCCACTGTTACCTGCCCC 480
H               TCGGCGGGTCTGAGTCTGGGCTATGACCCTGACTCGGGCCACTCCACTGTTACCTGCCCC 480
P               TCGGCGGGTCTGAGTCTGGGCTATGACCCTGACTCAGGCCACTCCACTGTTACCTGCCCC 480
                *********************************** ************************
KC894598.1      AGATGCAGCAGCCACATCAACACCGTCCGCATACGCATCTCTGGCAGCAGCGTGGGGTGG 540
H               AGATGCAGCAGCCACATCAACACCGTCCGCATACGCATCTCTGGCAGCAGCGTGGGGTGG 540
P               AGATGCAGCAGCCACATCAACACCGTCCGCATACGCATCTCTGGCAGCAGCGTGGGGTGG 540
                ************************************************************
KC894598.1      TTGATCCAACTCTTCCACAAAAGAATCGAGTCTTCGCTCCAAAACTCCATGACCAGCAAG 600
H               TTGATCCAACTCTTCCACAAAAGAATCGAGTCTTCGCTCCAAAACTCCATGACCAGCAAG 600
P               TTGATCCAACTCTTCCACAAAAGAATCGAGTCTTCGCTCCAAAACTCCATGACCAGCAAG 600
                ************************************************************
KC894598.1      ATCTGCGAGGTGGTGACTAGTACTGTGTCCTCCAAGCTGCAACCT      645
H               ATCTGCGAGGTGGTGACTAGTACTGTGTCCTCCAAGCTGCAACCT      645
P               ATCTGCGAGGTGGTGACTAGTACTGTGTCCTCCAAGCTGCAACCT      645
                *********************************************
```

Figure S13: Comparison of healthy and pneumonia-affected Baladi goats using GenBank-sourced reference sequence gb|KC894598.1| and the *BP1* gene (645-bp) demonstrating DNA sequence alignment.

```
XM_018048527.1      AACCGCAATACATGGTGCTGGTCCCCTCCCTGCTCCGTACCAGAATCCCTGAGAAGGGTT      60
H                   AACCGCAATACATGGTGCTGGTCCCCTCCCTGCTCGGTACCAGAATCCCTGAGAAGGGTT      60
P                   AACCGCAATACATGGTGCTGGTCCCCTCCCTGCTCCGTACCAGAATCCCTGAGAAGGGTT      60
                    ********************************** *************************

XM_018048527.1      GCCTCCTTCTGAGCCACCTGAATGAGACGGTGACTGTCAGCGCCTCCTTGGAGTCCGTCA      120
H                   GCCTCCTTCTGAGCCACCTGAATGAGACGGTGACTGTCAGCGCCTCCTTGGAGTCCGTCA      120
P                   GCCTCCTTCTGAGCCACCTGAATGAGACGGTGACTGTCAGCGCCTCCTTGGAGTCCGTCA      120
                    ************************************************************

XM_018048527.1      GAGAGAACAGGAGCCTCTTCACTGATGTGGTGGCAGAGAAGGACTTATTCCACTGTGTCT      180
H                   GAGAGAACAGGAGCCTCTTCACTGATGTGGTGGCAGAGAAGGACTTATTCCACTGTGTCT      180
P                   GAGAGAACAGGAGCCTCTTCACTGATGTGGTGGCAGAGAAGGACTTATTCCACTGTGTCT      180
                    ************************************************************

XM_018048527.1      CCTTCAGCCTCCCAAGATCTCCAACCAGTCAAGAGGTAATGTTCCTCACCATCCAAGTTA      240
H                   CCTTCAGCCTCCCAAGATCTCCAACCAGTCAAGAGGTAATGTTCCTCACCATCCAAGTTA      240
P                   CCTTCAGCCTCCCAAGATCTCCAACCAGTCAAGAGGTAATGTTCCTCACCATCCAAGTTA      240
                    ************************************************************

XM_018048527.1      AAGGACCAACCCAAGAATTCAAGAAGCGGACCACAGTGTTGGTTAAAAATGAAGAGAGCC      300
H                   AAGGACCAACCCAAGAATTCAAGAAGCGGACCACAGTGTTGGTTAAAAATGAAGAGAGCC      300
P                   AAGGACCAACCCAAGAATTCAAGAAGCGGACCACAGTGTTGGTTAAAAATGAAGAGAGCC      300
                    ************************************************************

XM_018048527.1      TGGTCTTTGTCCAGACAGACAAACC   325
H                   TGGTCTTTGTCCAGACAGACAAACC   325
P                   TGGTCTTTGTCCAGACAGACAAACC   325
                    *************************
```

Figure S14: Comparison of healthy and pneumonia-affected Baladi goats using GenBank-sourced reference sequence gb|XM_018048527.1| and the *A2M* gene (325-bp) demonstrating DNA sequence alignment.

```
XM_013962543.2      CTACTCACCTGGGAAGCTTCTCCTGGAAAACGAGATGCCTGTCAACAGCACTGCCGTGTC      60
H                   CTACTCACCTGGGAAGCTTCTCCTGGAAAACGAGATGCCTGTCAACAGCACTGCCGTGTC      60
P                   CTACTCACCTGGGAAGCTTCTCCTGGAAAACGAGATGCCTGTCAACAGCACTGCCGTGTC      60
                    ************************************************************
XM_013962543.2      CTTGACCAGTGTCACCTACATCACTGTGGAGATCCTCATCGGGCTCTGCGCCATAGTGGG      120
H                   CTTGACCAGTGTCACCTACATCACTGTGGAGATCCTCATCGGGCTCTGCGCCATAGTGGG      120
P                   CTTGACCAGTGTCACCTACATCACTGTGGAGATCCTCATCGGGCTCTGCGCCATAGTGGG      120
                    ************************************************************
XM_013962543.2      CAACGTGCTGGTCATCTGGGTGGTCAAGCTGAACCCCAGCCTGCAGACCACCACCTTCTA      180
H                   CAACGTGCTGGTCATCTGGGTGGTCAAGCTGAACCCCAGCCTGCAGACCACCACCTTCTA      180
P                   CAACGTGCTGGTCATCTGGGTGGTCAAGCTGAACCCCAGCCTGCAGACCACCACCTTCTA      180
                    ************************************************************
XM_013962543.2      TTTCATTGTCTCCCTAGCCCTGGCTGACATTGCTGTCGGGGTGCTGGTTATGCCTCTGGC      240
H                   TTTCATTGTCTCCCTAGCCCTGGCTGACATTGCTGTCGGGGTGCTGGTTATGCCTCTGGC      240
P                   TTTCATTGTCTCCCTAGCCCTGGCTGACATTGCTGTCGGGGTGCTGGTTATGCCTCTGGC      240
                    ************************************************************
XM_013962543.2      CATTGTCATCAGCCTGGGTGTCACAATCCACTTTTATAGCTGCCTTTTCATGACCTGCTT      300
H                   CATTGTCATCAGCCTGGGTGTCACAATCCACTTTTATAGCTGCCTTTTCATGACCTGCTT      300
P                   CATTGTCATCAGCCTGGGTGTCACAATCCACTTTTATAGCTGCCTTTTCATGACCTGCTT      300
                    ************************************************************
XM_013962543.2      GATGCTGATCTTCACCCACGCATCCATCATGTCCTTGCTAGCCATTGCTGTGGACCGATA      360
H                   GATGCTGATCTTCACCCACGCATCCATCATGTCCTTGCTAGCCATTGCTGTGGACCGATA      360
P                   GATGCTGATCTTCACCCACGCATCCATCATGTCCTTGCTAGCCATTGCTGTGGACCGATA      360
                    ************************************************************
XM_013962543.2      CCTGCGGGTCAAGCTCACAGTCAGATCCAGGATTCCTAAGGCCCCTGGGTCCATCTGGTC      420
H                   CCTGCGGGTCAAGCTCACAGTCAGATCCAGGATTCCTAAGGCCCCTGGGTCCATCTGGTC      420
P                   CCTGCGGGTCAAGCTCACAGTCAGATACAGGATTCCTAAGGCCCCTGGGTCCATCTGGTC      420
                    ************************** *********************************
XM_013962543.2      ATCCCAGTTGAAGATTCACTTCTTTCCAGTCATGAGGCTCTTCATTCTGCTCTCTTTGAC      480
H                   ATCCCAGTTGAAGATTCACTTCTTTCCAGTCATGAGGCTCTTCATTCTGCTCTCTTTGAC      480
P                   ATCCCAGTTGAAGATTCACTTCTTTCCAGTCATGAGGCTCTTCATTCTGCTCTCTTTGAC      480
                    ************************************************************
XM_013962543.2      TGTCTTTTCAGATGCCATGGTCATGGACGAGAAGGTCAAGG      521
H                   TGTCTTTTCAGATGCCATGGTCATGGACGAGAAGGTCAAGG      521
P                   TGTCTTTTCAGATGCCATGGTCATGGACGAGAAGGTCAAGG      521
                    *****************************************
```

Figure S15: Comparison of healthy and pneumonia-affected Baladi goats using GenBank-sourced reference sequence gb|XM_013962543.2| and the *ADORA3* gene (521-bp) demonstrating DNA sequence alignment.

```
KJ001638.1      ATGTGCGCACAAGATGCTTTTCAGACACAGAGAAGCCAGCTGGTGGAGTTGCTGGTCTCG 60
H               ATGTGCGCACAAGATGCTTTTCAGACACAGAGAAGCCAGCTGGTGGAGTTGCTGGTCTCG 60
P               ATGTGCGCACAAGATGCTTTTCAGACACAGAGAAGCCAGCTGGTGGAGTTGCTGGTCTCG 60
                ************************************************************
KJ001638.1      GGGTCCCTGGAGAGCTTTGAGAGTATTGTGGACCGGCTGCTTTCCCGGGAAGTCCTCTCC 120
H               GGGTCCCTGGAGAGCTTTGAGAGTATTGTGGACCGGCTGCTTTCCCGGGAAGTCCTCTCC 120
P               GGGTCCCTGGAGGGCTTTGAGAGTATTGTGGACCGGCTGCTTTCCCGGGAAGTCCTCTCC 120
                ************ ***********************************************
KJ001638.1      TGGGAGGACTATGAGGGGCTTAGCCTCGTGGGCCAGCCCATCTCCCACTTGGCCAGGCGC 180
H               TGGGAGGACTATGAGGGGCTTAGCCTCGTGGGCCAGCCCATCTCCCACTTGGCCAGGCGC 180
P               TGGGAGGACTATGAGGGGCTTAGCCTCGTGGGCCAGCCCATCTCCCACTTGGCCAGGCGC 180
                ************************************************************
KJ001638.1      CTCCTGGACACCATCTGGAATAAGGGTGCTTGGGGCTGTGAACAACTGACTGCAGCTGTG 240
H               CTCCTGGACACCATCTGGAATAAGGGTGCTTGGGGCTGTGAACAACTGACTGCAGCTGTG 240
P               CTCCTGGACACCATCTGGAATAAGGGTGCTTGGGGCTGTGAACAACTGACTGCAGCTGTG 240
                ************************************************************
KJ001638.1      TGGGAGGCCCAGGCCGACAGCCAGCCCCCCGAGCTTCCCAGCTCCTGGGACCCCCACTCA 300
H               TGGGAGGCCCAGGCCGACAGCCAGCCCCCCGAGCTTCCCAGCTCCTGGGACCCCCACTCA 300
P               TGGGAGGCCCAGGCCGACAGCCAGCCCCCCGAGCTTCCCAGCTCCTGGGACCCCCACTCA 300
                ************************************************************
KJ001638.1      CCCCACCCAGCCCGTGACCTGCAGAGTCATCGGCCAGCCATCGTCAGGAGACTCTACGGC 360
H               CCCCACCCAGCCCGTGACCTGCAGAGTCATCGGCCAGCCATCGTCAGGAGACTCTACGGC 360
P               CCCCACCCAGCCCGTGACCTGCAGAGTCATCGGCCAGCCATCGTCAGGAGACTCTACGGC 360
                ************************************************************
KJ001638.1      CACGTGGAGGGTGTGCTGGACCTGACACAGCAGC          394
H               CACGTGGAGGGTGTGCTGGACCTGACACAGCAGC          394
P               CACGTGGAGGGTGTGCTGGACCTGACACAGCAGC          394
                **********************************
```

Figure S16: Comparison of healthy and pneumonia-affected Baladi goats using GenBank-sourced reference sequence gb|KJ001638.1| and the *CARD15* gene (394-bp) demonstrating DNA sequence alignment.

```
JQ308793.1      ACTCCTGGGAAGGATAAGCCCGACCTGCCCACATGGAAGAGGAATTTCCGGTCTGCCCTG 60
H               ACTCCTGGGAAGGATAAGCCCGACCTGCCCACATGGAAGAGGAATTTCCGGTCTGCCCTG 60
P               ACTCCTGGGAAGGATAAGCCCGACCTGCCCACATGGAAGAGGAATTTCCGGTCTGCCCTG 60
                ************************************************************

JQ308793.1      AACCGGAAGGAAGTGTTGCGTTTAGCGGAGGACCACAGCAAGGACTCCCAAGACCCGCAC 120
H               AACCGGAAGGAAGTGTTGCGTTTAGCGGAGGACCACAGCAAGGACTCCCAAGACCCGCAC 120
P               AACCGGAAGGAAGTGTTGCGTTTAGCGGAGGACCACAGCAAGGACTCCCAAGACCCGCAC 120
                ************************************************************

JQ308793.1      AAGATCTATGAGTTTGTGAACTCAGGGGTCAGGGACATCCCTGAGACAGATACCGCTCAA 180
H               AAGATCTATGAGGTTGTGAACTCAGGGGTCAGGGACATCCCTGAGACAGATACCGCTCAA 180
P               AAGATCTATGAGTTTGTGAACTCAGGGGTCAGGGACATCCCTGAGACAGATACCGCTCAA 180
                ************ ***********************************************

JQ308793.1      GACAATGGCAGACACAGTACCTCTGATACCCAGGAAGACATTGTGCAGAAGTTACTGAGT 240
H               GACAATGGCAGACACAGTACCTCTGATACCCAGGAAGACATTGTGCAGAAGTTACTGAGT 240
P               GACAATGGCAGACACAGTACCTCTGATACCCAGGAAGACATTGTGCAGAAGTTACTGAGT 240
                ************************************************************

JQ308793.1      GACATGGACTTGAGCCCAGAAGGAGGGCCCTCCAATCTGACTATGACCTCTGAGAACCCC 300
H               GACATGGACTTGAGCCCAGAAGGAGGGCCCTCGAATCTGACTATGACCTCTGAGAACCCC 300
P               GACATGGACTTGAGCCCAGAAGGAGGGCCCTCCAATCTGACTATGACCTCTGAGAACCCC 300
                ******************************** ***************************

JQ308793.1      CCTCAGCTCTTACTGAGCCCCGAATCAGACATCCCTGCTCTTTGCCCAAACTCGGGACTC 360
H               CCTCAGCTCTTACTGAGCCCCGAATCAGACATCCCTGCTCTTTGCCCAAACTCGGGACTC 360
P               CCTCAGCTCTTACTGAGCCCCGAATCAGACATCCCTGCTCTTTGCCCAAACTCGGGACTC 360
                ************************************************************

JQ308793.1      TCTGAAAACCCCCTGAAGCAGCTGTTGGCGAACGAGGAAGATTGGGAGTTCGAGGTGACT 420
H               TCTGAAAACCCCCTGAAGCAGCTGTTGGCGAACGAGGAAGATTGGGAGTTCGAGGTGACT 420
P               TCTGAAAACCCCCTGAAGCAGCTGTTGGCGAACGAGGAAGATTGGGAGTTCGAGGTGACT 420
                ************************************************************

JQ308793.1      GCCTTCTACCGGGGCTGTCAAGTCTTCCAGCAGACTGTCTTCTGCCCT   468
H               GCCTTCTACCGGGGCTGTCAAGTCTTCCAGCAGACTGTCTTCTGCCCT   468
P               GCCTTCTACCGGGGCTGTCAAGTCTTCCAGCAGACTGTCTTCTGCCCT   468
                ************************************************
```

Figure S17: Comparison of healthy and pneumonia-affected Baladi goats using GenBank-sourced reference sequence gb|JQ308793.1|and the *IRF3* gene (468-bp) demonstrating DNA sequence alignment.

```
XR_001917340.1      CTGAGATCAGGAATGTGAACGGGGTGGACCGCCTCTGTGGCCTGCACCGGGAGGAGGCCA      60
H                   CTGAGATCAGGAATGTGAACGGGGTGGACCGCCTCTGTGGCCTGCACCGGGAGGAGGCCA      60
P                   CTGAGATCAGGAATGTGAACGGGGTGGACCGCCTCTGTGGCTTGCACCGGGAGGAGGCCA      60
                    ***************************************** ******************

XR_001917340.1      CGGTGTTCTGCCAGGAGCTGGGGTGAGGCCCTGCCCTGCAGGTCCCCCGCCAAGATGGCA      120
H                   CGGTGTTCTGCCAGGAGCTGGGGTGAGGCCCTGCCCTGCAGGTCCCCCGCCAAGATGGCA      120
P                   CGGTGTTCTGCCAGGAGCTGGGGTGAGGCCCTGCCCTGCAGGTCCCCCGCCAAGATGGCA      120
                    ************************************************************

XR_001917340.1      GTGTCACCAGGAAGTACATGACCTGTAGGGGCGACGAGCTGACCATCCGGAACTGCAGAC      180
H                   GTGTCGCCAGGAAGTACATGACCTGTAGGGGCGACGAGCTGACCATCCGGAACTGCAGAC      180
P                   GTGTCACCAGGAAGTACATGACCTGTGGGGGCGACGAGCTGACCATCCGGAACTGCAGAC      180
                    ***** ******************** *********************************

XR_001917340.1      TGAACAAGTTCCGCAGCGGCTGCGACTTCCAGCGAGATGCCCAGGTGGTCTGCTCAGGGC      240
H                   TGAACAAGTTCCGCAGCGGCTGCGACTTCCAGCGAGATGCCCAGGTGGTCTGCTCAGGGC      240
P                   TGAACAAGTTCCGCAGCGGCTGCGACTTCCAGCGAGATGCCCAGGTGGTCTGCTCAGGGC      240
                    ************************************************************

XR_001917340.1      ACACGGAGGCCCGGCTGGCGGGTGGCGGGCACTCCTGTGCTGGGCGCCTGGAGGTGAGGC      300
H                   ACACGGAGGCCCGGCTGGCGGGTGGCGGGCACTCCTGTGCTGGGCGCCTGGAGGTGAGGC      300
P                   ACACGGAGGCCCGGCTGGCGGGTGGCGGGCACTCCTGTGCTGGGCGCCTGGAGGTGAGGC      300
                    ************************************************************

XR_001917340.1      GTGGCCTGACCTGGGGCACCGTCTGTGACGCTGACCTGGACCTGGCCACTGCCCACGTGG      360
H                   GTGGCCTGACCTGGGGCACCGTCTGTGACGCTGACCTGGACCTGGCCACTGCCCACGTGG      360
P                   GTGGCCTGACCTGGGGCACCGTCTGTGACGCTGACCTGGACCTGGCCACTGCCCACGTGG      360
                    ************************************************************

XR_001917340.1      TGTGCCGGGAGCTGCAGTGTGGTGTGGCCGTGTCCACACCCCAGGGCGCCCACTTTGGCC      420
H                   TGTGCCGGGAGCTGCAGTGTGGTGTGGCCGTGTCCACACCCCAGGGCGCCCACTTTGGCC      420
P                   TGTGCCGGGAGCTGCAGTGCGGTGTGGCCGTGTCCACACCCCAGGGCGCCCACTTTGGCC      420
                    ******************* ****************************************

XR_001917340.1      AGGGCTCGGGGCTCGTGTGGACCGAGGCCTTCCGCGGGCAACGAGTCCCTGCTGT  475
H                   AGGGCTCGGGGCTCGTGTGGACCGAGGCCTTCCGCGGGCAACGAGTCCCTGCTGT  475
P                   AGGGCTCGGGGCTCGTGTGGACCGAGGCCTTCCGCGGGCAACGAGTCCCTGCTGT  475
                    *******************************************************
```

Figure S18: Comparison of healthy and pneumonia-affected Baladi goats using GenBank-sourced reference sequence gb|XR_001917340.1| and the *SCART1* gene (475-bp) demonstrating DNA sequence alignment.
